# Isolation and Identification of Culturable Bacteria from South China Seawater and Preliminary Screening of Marine Biocontrol Bacteria

**DOI:** 10.3390/microorganisms11122933

**Published:** 2023-12-06

**Authors:** Limei Guan, Hongxiu Wang, Junhui Chen, Feiying Yang, Jian Yang, Jianghuai Li, Liang Jin

**Affiliations:** 1 Institute of Biological Resources, Jiangxi Academy of Sciences, Nanchang 330029, China; glmnh@126.com (L.G.); allenchen0426@gmail.com (J.C.); 18059141865@163.com (F.Y.); jemappelleyangjian@zju.edu.cn (J.Y.); jeremy_leakey@sina.com (J.L.); 2 Institute of Microbiology, Jiangxi Academy of Sciences, Nanchang 330029, China; wanghongxiu0624@163.com

**Keywords:** antagonistic bacteria, antimicrobial fungi, bacterial diversity, culturable bacteria, marine water, phytopathogenic fungi

## Abstract

Marine microorganisms have evolved special metabolic pathways to produce numerous bioactive substances with novel structures and unique functions. This study analyzed the diversity of culturable bacteria in marine water samples from the South China Sea and screened the isolated bacteria with pathogenic fungi. A total of 200 culturable strains of 72 different bacteria were obtained from 56 water samples from the South China Sea. They belonged to three phyla and four classes, namely Gammaproteobacteria, Alphaproteobacteria, Bacilli and Actinomycetia. Bacilli was the dominant class, comprising up to 59.72%, followed by Gammaproteobacteria (20.83%). *Bacillus*, *Pseudomonas*, *Paenibacillus* and *Rhizobium* were the most dominant genera. Among these strains, HY-88 and HY-91 encoding *Bam*C, *Fen*B and *PKS*I genes were selected and identified as *Bacillus subtilis*. The respective inhibition rates of the HY-88 caused by plate confrontation against *Magnaporthe grisea*, *Fusarium oxysporum*, *Botrytis cinerea*, *anthrax* and *Botrytis cinerea* were 90.91%, 54.29%, 52.17% and 51.72%, in comparison with HY-91 86.36%, 48.57%, 47.83% and 34.48%. In addition, the supernatant of HY-88 showed a lesion inhibition rate of 74.5%, which was significantly higher than HY-91 (60.55%). In addition, HY-88 and HY-91 showed strong antifungal activity to *Colletotrichum viniferum* on detached Shine Muscat grapes. Tolerance tests showed that the HY-88 and HY-91 grew at 10–40 °C, 7–10% NaCl and pH 3-11. HY-88 and HY-91 could inhibit various fungal plant diseases, which lays a foundation for the development of new biopesticides.

## 1. Introduction

The South China Sea is in the tropical and subtropical regions of the southern part of the Asian continent, with year-round high temperatures, high humidity and high salinity. As an important part of the South China Sea marine ecosystem, the microbial community has received extensive attention for its marine water characteristics, community structure and bacterial resources [1,2,3]. During the long-term evolution of microorganisms in the South China Sea’s marine ecosystem, their ecological characteristics, bacterial community structure and functional diversity have adapted to the extreme environments [4,5].

International research has paid special attention to marine microbial natural products. In 2021, Carroll et al. [6] summarized the latest reports on marine natural products and found that 55% of them were derived from marine microorganisms. Researchers have obtained new active substances from marine microorganisms, including anticancer, antifouling, antibacterial and antifungal activities [7].

Phytopathogenic fungi can cause disease in all kinds of plants, such as the passion fruit leaf blight caused by *Nigrospora sphaerica* WYR007 [8] and *Botrytis cinerea*, one of the most important fruit and vegetable phytopathogenic fungi [9]. Rotten fruits or vegetables affected by phytopathogenic fungi will produce some secondary toxic metabolites, which are probably harmful to human and animal health [10]. *Magnaporthe grisea* can cause serious disease in many species of the grass family, including rice, wheat and barley [11], and have a huge impact, causing serious economic losses for local growers.

Some bacteria also show strong biological activities with a wide antibacterial spectrum and abundant active substances but no resistance and pollution [12,13,14]. At present, plant disease control heavily relies on chemical control, but excessive use of chemical pesticides harms the ecological environment and agricultural products and accelerates the mutation of some strains to develop resistance to pesticides [15]. In addition, the breeding cycle of disease-resistant varieties is long, and resources are limited. Among all control methods, biological control is a sustainable strategy, and is increasingly receiving the most attention as an important means to curb plant diseases and improve crop yields [16]. In recent years, scientists have screened marine bacteria for use in agricultural fungal disease control and biomedicine [17,18,19]. The tolerance, physiological and biochemical reactions, metabolic pathways and products of marine microorganisms in the environment will change correspondingly with the influence of the marine environment, producing bioactive substances with unique functions and novel structures [20,21,22]. In recent years, the search for new microbial strains and bioactive substances with special functions from the ocean has become a research hotspot [23]. Ma [24] obtained an antifungal compound, SPM5C-1, showing strong activity against blast fungus. After being sprayed on rice, it can significantly reduce the occurrence of rice blasts, and can also improve rice grain yield. Marine natural product antimycin A inhibits mycelial growth, conidiogenesis, germination and harmful morphological changes, and inhibits wheat blast [25]. The research direction of developing new strains and active substances of marine microorganisms for agricultural production is innovative and has great application prospects, which will help to explore the functional role of marine microorganisms regarding the antagonism mechanism of plant diseases.

In this study, marine water samples from the South China Sea were used to analyze the diversity of culturable bacteria composition. Marine biocontrol bacteria with strong inhibitory effects on plant pathogenic fungi were screened, which provides a research basis and a theoretical basis for the next development of new biological pesticides.

## 2. Materials and Methods

### 2.1. Source of Pathogenic Fungi and Preparation of Culture-Medium

*Magnaporthe grisea* Guy11 was obtained from the National Key Laboratory of Crop Genetic Improvement, Huazhong Agricultural University. *Botrytis Cinerea*, *Colletotrichum viniferum* and *Fusarium oxyporum* were obtained from the Fruit Research Institute, Chinese Academy of Agricultural Sciences. All culturable marine bacteria were grown on R2A medium (HKM Huankai microorganism) [26]. All fungi were grown on potato glucose medium agar (Hangzhou Best Biotechnology Co., Ltd., Hangzhou, China).

### 2.2. Water Sample Collection

A total of 56 water samples from the South China Sea were collected in May and June 2019. According to the depth of the survey station and its layer distribution, the survey section was set. In principle, a minimum of three 50 m sections were set along the isobath at the depth ranges of 2–4 m, 6–8 m and 10–15 m. The sections were parallel to each other, more than 50 m apart, and located at different water depths. The longitude range of water sample collection was 112°52′40″–114°17′10″ E and the latitude range was 9°32′9″–10°56′28″ N.

### 2.3. Isolation of Culturable Bacteria

Under aseptic conditions, 1 mL marine water samples were added to 9 mL sterile water to dilute 10^−1^ and successively diluted to 10^−2^–10^−6^ with sterile water via a step dilution method. A 500 μL sample of each dilution was coated and separated on seawater R2A medium plates [27], and each dilution was repeated three times. Colonies with different growth traits were cultured in a constant temperature incubator at 37 °C for 1–3 days. After several generations of purification, the single colony was picked up and cultured for further experiments.

### 2.4. Genomic DNA Extraction and Bacterial 16S rRNA Gene Amplification

The DNA extraction kit (TIANGEN) was used to extract the genomic DNA of each bacterium. Taking the DNA extracted from each bacterium as a template, the full length of the 16S rRNA fragment (about 1.5 kb) was amplified by bacterial general primer 27F/1492R. The PCR products of 200 strains were obtained via 1% agarose gel electrophoresis from 10 μL of PCR products and were then sent to Shanghai Shenggong Bioengineering Co., Ltd. (Shanghai, China) for sequencing. The primer sequences are shown in Table 1.

### 2.5. Phylogenetic and Diversity Analysis of Culturable Bacteria

The obtained sequences were submitted to the Ribosomal Database Project II (RDP) database, and the chimeras were detected using the online detection tool CHECK-CHIMERA. The valid sequences after removing the chimeras were then searched for highly homologous sequences using the BLASTN program of GenBank. Multi-sequence matching was performed using the ClustalX program, then the phylogenetic tree was constructed using the Neighbor-Joining (NJ) method in the MEGA 4.0 program and the Jukes-Cantor calculation model. It is glorified by the website ITOL [28]. The bootstrap value was set to 1000. The serial numbers were MZ895383–MZ895454 in GenBank. The species abundance map was analyzed with R software, all calculations were conducted, and graphs were drawn in R (v.3.6.3) using “networkD3”, “dplyr”, “readxl” and “extrafont” packages 45 [29,30,31,32]. Diversity analysis of culturable bacteria in different water depths was conducted with GraphPad Prism 9.

### 2.6. PSKI Gene and Antimicrobial Gene Screening

The amplification procedures were as follows: 98 °C, 2 min; 98 °C, 10 s; 58 °C, 15 s; 72 °C, 20 s. There were 32 cycles, which were then extended at 72 °C for 10 min. The PCR products were detected via 1% agarose gel electrophoresis synthesized by Shanghai Shenggong Bioengineering Co., Ltd. The primer sequences are shown in Table 1.

**Table 1:** Primers for PCR amplification [33,34].

Gene	Function	Primer	Primer Sequence	Product Size
*16S r*RNA	species identification	27F/ 1492R	5′-AGAGTTTGATCCTGGCTCAG-3′ 5′-AAG TCGTAACAAGGTARCCGTA-3′	1500 bp
*PSKI*	polyketide synthase	KSF*/* KSR	5′-GCGATGGATCCNCAGCAGCG-3′ 5′-GTGCCGGTNCCGTGNGYYTC-3′	750 bp
*bam*C	bacillomycin	*bam*C1F*/* *bam*C1R	5′-AGTAAATGAACGCGCCAATC-3′ 5′-CCCTCTCCTGCCACATAGAG-3′	975 bp
*fen*B	Photigenin synthetase	*fen*BF*/* *fen*BR	5′-CTATAGTTTGTTGACGGCTC-3′ 5′-CAGCACTGGTTCTTGTCGCA-3′	1400 bp
*itu*D	Istilisin synthetase	*itu*D2F/ *itu*D2R	5′-CGCGATCCATGAACAATCTTGCCTTTTTA-3′ 5′-CCGCTCGAGTTATTTTAAAATCCGCAATT-3′	1200 bp

### 2.7. Determination of the Antifungal Activity against Phytopathogens

Using the plate confrontation method, the strains with genes related to fungistatic substances were inoculated on seawater R2A medium plates, activated and cultured at 30 °C for 12–16 h. Single colonies were selected on the LB liquid medium and shaken at 30 °C with 180 rpm/min for 24 h as seed liquid. The pathogenic fungal blocks of the tested plants were then inoculated into the center of the PDA plate, and the antagonistic bacteria were inoculated for four weeks. Mycelial growth inhibition rate = (control colony diameter confrontation plate colony diameter)/control colony diameter × 100%. We then antagonized the antifungal effect of the fermentation broth supernatant on plant pathogens. Using the plate confrontation method, the initially screened bacteriostatic strains were inoculated on seawater R2A medium plate, activated and cultured at 30 °C for 12–16 h and single colonies were selected in the LB liquid medium. After being shaken and cultured at 30 °C for 24 h at 180 rpm/min, the fermentation broth supernatant was centrifuged at 12,000× *g*/min for later use. The pathogenic fungal blocks of the tested plants were inoculated in the center of the PDA agar plate, and the supernatant of the fermentation broth of the antagonistic bacteria was inoculated. PDA plates containing only patches of pathogenic fungi were inoculated as controls. To study the influence of strain fermentation broth supernatant on the pathogen mycelia, the mycelia at the edge of the inhibition zone were selected, and the morphological changes of mycelia were observed under a light microscope (CX33RTFS2, OLYMPUS).

### 2.8. Antifungal Activity Assays of the Strains

The stab wound method was used for the evaluation of antifungal activity against *Colletotrichum viniferum* [35,36]. Shine Muscat grape was picked following local cultivation. Healthy fruit was free from physical injuries and homogeneous in size and maturity. Prior to the experiments, the grape surface was washed with tap water, disinfected with 70% ethanol for 1 min, rinsed twice with sterile water and air-dried on sterile filter paper on a bench. A cross wound with a diameter of 3 mm was stabbed in the middle of the grape with a sterile needle. Then, a 3 mm square lump of *Colletotrichum viniferum* was stuck to the wound for 24 h. The sterile PDA was added as a negative control. A 10 µL volume of HY-88 or HY-91 fermentation broth at 10^8^ CFU/mL was injected into the wound. Every treatment contained 10–12 grapes and 3 replicates were conducted. The experiment was conducted twice at different times. Treated grapes were stored in a plastic crisper, sprayed with sterilized water daily and kept in a 22 °C incubator. After 7 days of culturing, lesion diameters were measured to evaluate the biocontrol efficacy. The cross method was used to calculate the diameter of the lesions, and the inhibition rate of the lesions in each lesion treatment was calculated.

$$lesion\;inhibition\;rate\;\left(\%\right)=\frac{lesion\;diameter\;of\;the\;CV\;group\;-\;diameter\;of\;the\;treatment\;group}{lesion\;diameter\;of\;the\;CV\;group}\times 100$$

CV group means *Colletotrichum viniferum-*treated group.

### 2.9. Tolerance Tests of Strains

Growth temperature range experiment: A single colony was selected and inoculated on R2A inclined surface medium then cultured at 30 °C for 12–16 h. Single colonies were selected in the 5 mL LB liquid medium. After they had been shaken and cultured at 0 °C, 4 °C, 10 °C, 15 °C, 30 °C, 40 °C, 50 °C, 60 °C and 70 °C for 14–16 h at 180 rpm/min, UV spectrophotometry OD600 was measured.

Salt tolerance test: NaCl was added at a series of concentrations (0, 1%, 3%, 5%, 7%, 9% and 10%) in R2A medium. A single colony was selected and cultured in a liquid medium with different NaCl concentrations for 14–16 h. UV spectrophotometry OD600 was observed and measured.

Acid and alkali resistance test: R2A medium with different pH values (1.0, 3.0, 5.0, 7.0, 9.0, 11.0, 13.0 and 14.0) was prepared, a single colony was inoculated in the prepared medium for 14–16 h and the UV spectrophotometric value OD600 was observed and measured.

### 2.10. Statistical Analysis

SPSS (version 21.0) was used to analyze the data. Data were subjected to analysis of variance (ANOVA) and the means were separated at the 5% significance level through Duncan Multiple Range Tests (DMRT). R software 3.5.2 was used to analyze the phylogenetic data and Prism primer 8.0.2 was used to draw the pictures. 

## 3. Results

### 3.1. Identification and Phylogenetic Analysis of Culturable Bacteria

**Figure 1:**
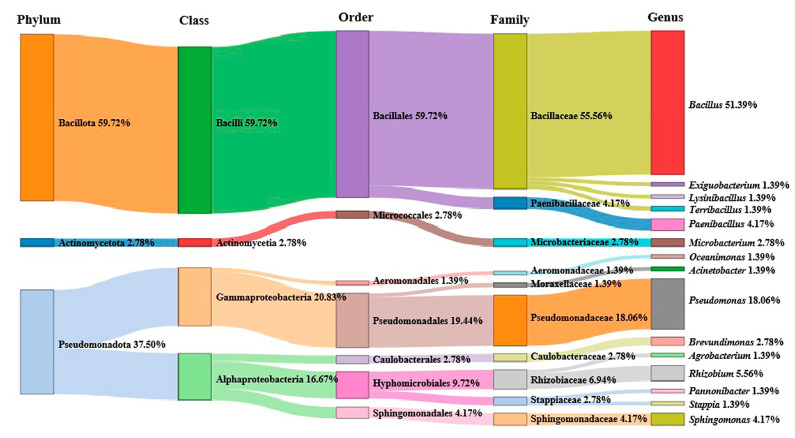
Proportion of different bacterial classes and genera in water samples from South China Sea.

**Figure 2:**
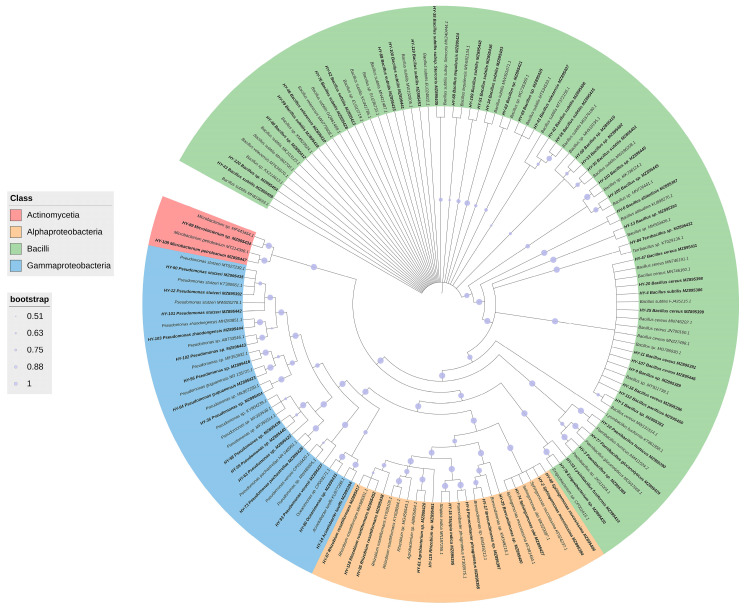
Proportion of different bacterial classes in water samples from South China Sea.

A total of 200 culturable bacterial 16S rRNA fragments were identified via PCR from 56 marine water samples from the South China Sea. After sequence analysis, 72 different bacterial strains belonging to 3 phyla, 4 classes, 7 orders, 10 families and 15 genera were obtained. Among them, *Bacillus* was the most abundant genus, accounting for up to 51.39% (Figure 1). The phylogenetic analysis results showed that 72 different bacteria belonged to four classes: Gammaproteobacteria, Alphaproteobacteria, Bacilli and Actinomycetia. Bacilli was the most dominant class (59.72%), followed by Gammaproteobacteria (20.83%) and Alphaproteobacteria (16.67%) (Figure 2).

### 3.2. Diversity Analysis of Culturable Bacteria in Different Water Depths

**Figure 3:**
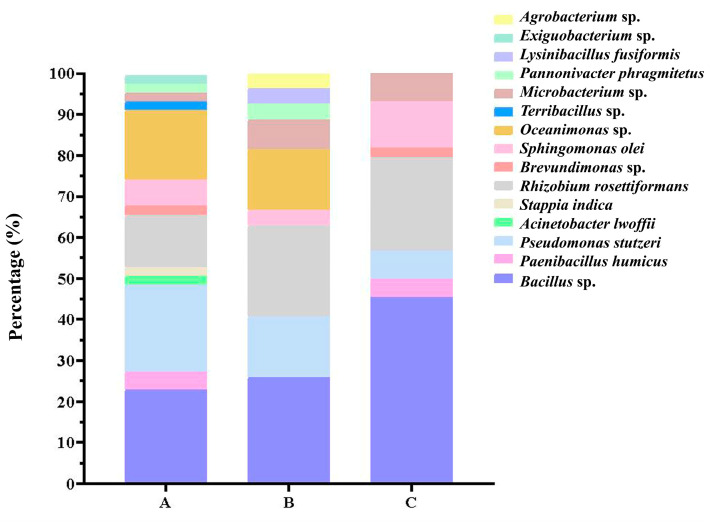
Distribution of cultivated microbes isolated at different depths in water samples from South China Sea. (A: 2–4 m; B: 6–8 m; C:10–15 m).

There was no significant difference between water depths. The average of 2–4 m, 6–8 m and 10–15 m was 2.47, 1.47 and 2.4 strains/sample, but the ANOVA result showed no significant difference between different water depths (*p* = 0.59). Multiple range tests also showed no significant differences between samples from different depths (all *p* > 0.5). *Bacillus* sp. was the most abundant species in all depths, especially at 10–15 m, accounting for up to 45.45% (Figure 3).

### 3.3. Detection and Analysis of Functional Genes BamC, fenB, ituD and PKSI

**Figure 4:**
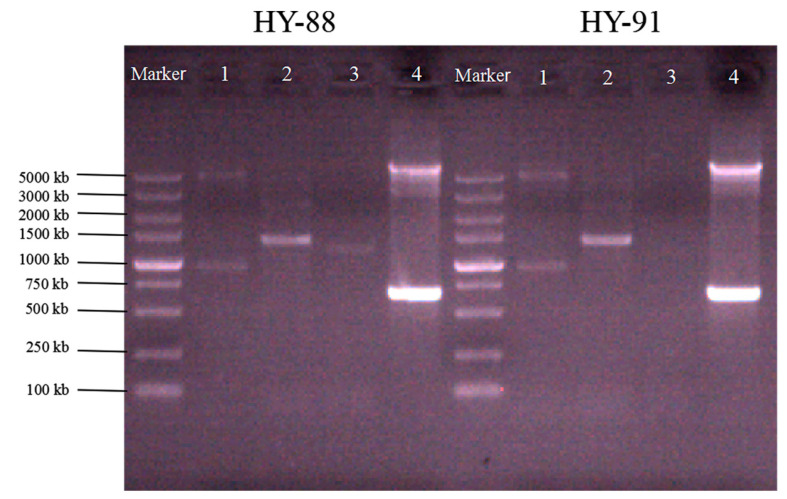
Functional analysis of the gene (1: *Bam*C; 2: *fen*B; 3: *itu*D; 4: *PKS*I).

A total of 18 strains with fungistatic genes were screened through sequence alignment (Figure 4). Two strains, HY-88 and HY-91, were identified using PCR amplification. The functional gene detection could amplify the polyketide compound synthesis gene PKSI with a target fragment of 750 bp, and the band was bright. At the same time, the two strains detected that the *fen*B gene involved in the synthesis of lipopeptide antibiotics had a band of 1400 bp, and the *Bam*C fragment size was 957 bp, which was weaker than that of the *PKS*I gene. HY-88 also detected a 1200 bp fragment of *itu*D, a gene involved in the synthesis of lipopeptide antibiotics, as shown in Figure 4. Subsequent fungal inhibition experiments mainly focused on the two strains HY-88 and HY-91 antagonists.

### 3.4. Phylogenetic Analysis of Strains HY-88 and HY-91

**Figure 5:**
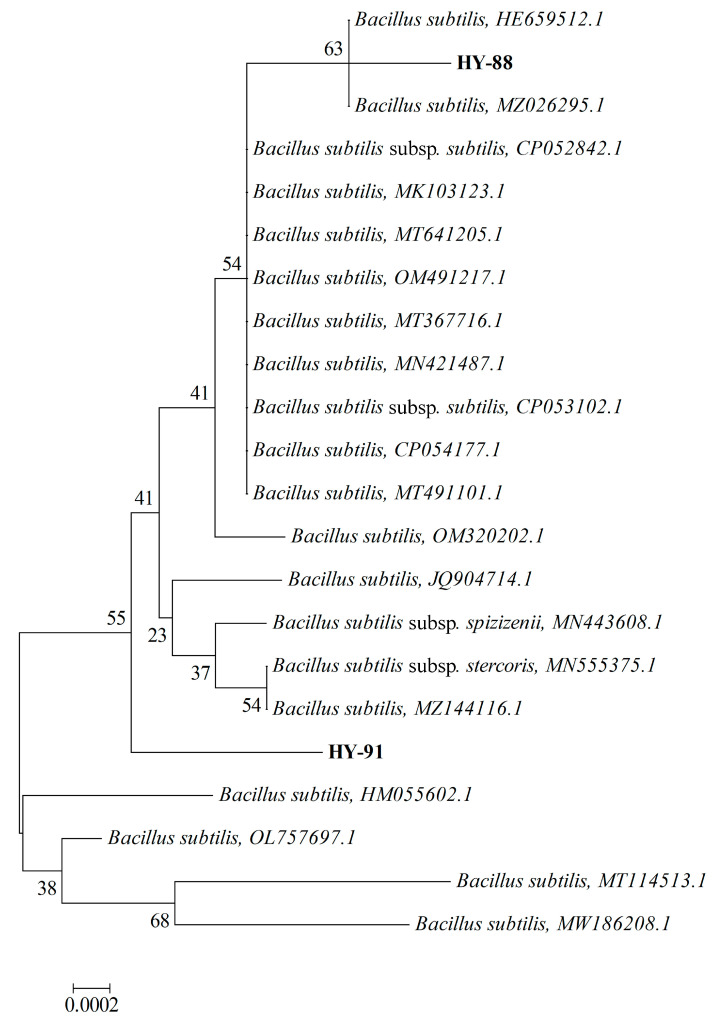
Phylogenetic tree showing the relationship between strain HY 88 and HY91. The tree was constructed using the neighbor-joining method based on the full-length 16S rDNA sequences. The scale bar represents a 10% sequence divergence and percentages of 1000 bootstrap re-samplings are shown at the nodes. The scale is 0.002.

The BLAST tool analysis results indicated that both HY-88 (MZ895433) and HY-91 (MZ895436) belonged to the species *Bacillus subtilis* (Figure 5). HY-88 had the closest homology with *Bacillus subtilis* (HE659512.1) and *Bacillus subtilis* (MZ026295.1), with 99.72% sequence identity. HY-91 was closely related to *Bacillus subtilis* (OL757697.1), with 99.93% sequence identity. The strains HY88 (MZ895433) and HY-91 (MZ895436) were in different outgroups.

### 3.5. Inhibitory Effect of HY-88 and HY-91 on Phytopathogenic Fungi

**Figure 6:**
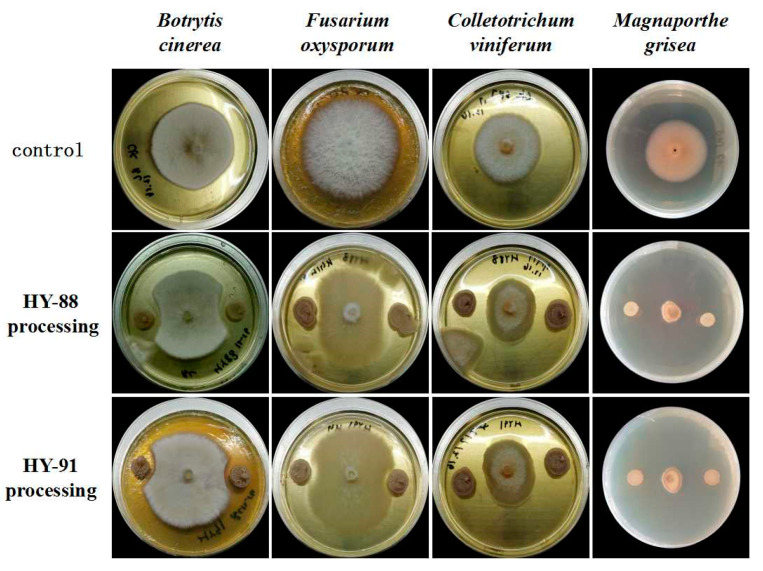
Antifungal effect of HY-88 and HY-91 against different phytopathogens.

Plate confrontation showed that HY-88 and HY99 were antagonistic to different phytopathogens (Figure 6). HY-88 and HY99 were most antagonistic to *Magnaporthe grisea* with inhibition rates of 90.91% and 80.34%. The inhibition rates of HY-88 against *Botrytis cinerea*, *Colletotrichum viniferum* and *Fusarium oxyporum* were 51.72%, 54.29% and 52.17%, respectively. Similarly, HY-91 showed an inhibition effect on *Botrytis cinerea*, *Colletotrichum viniferum* and *Fusarium oxyporum* with 34.48%, 48.57% and 47.83% inhibition rates, respectively (Table 2).

### 3.6. Inhibitory Activity of Colletotrichum viniferum on Detached Fruit of Shine Muscat Grape

**Figure 7:**
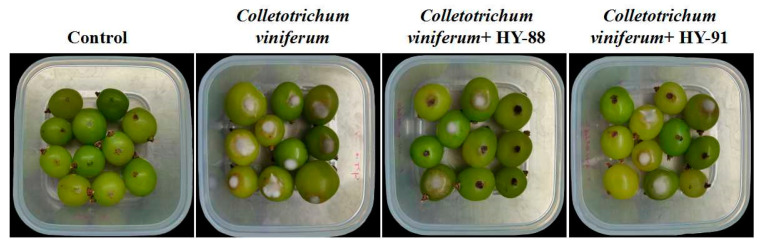
Pathogenic test of HY-88 and HY-91 against *Colletotrichum viniferum* on detached fruit of Shine Muscat grape.

Both HY-88 and HY-91 showed inhibitory activity of *Colletotrichum viniferum* on detached fruit of Shine Muscat grape (Figure 7). No infection was observed in the negative control group. The infection rate was 100% and the lesion diameters were 81.75 mm in the *Colletotrichum viniferum* group. The treatment of HY88 with a lesion inhibition rate of 74.5% was significantly lower than that of HY91 (60.55%, *p* < 0.05).

### 3.7. Antagonistic Effect of Supernatant of Fermentation Broth against Magnaporthe grisea

**Figure 8:**
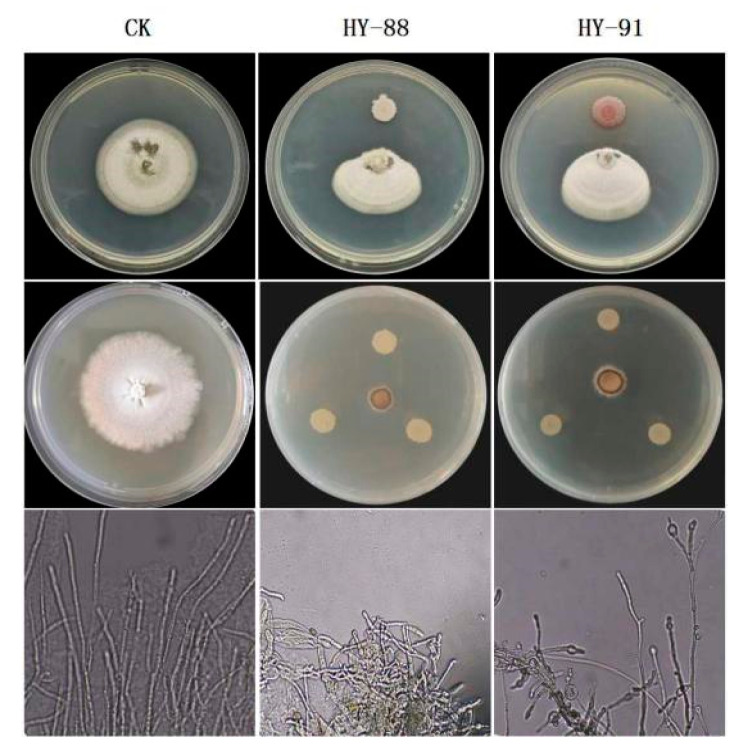
Inhibitory effects and mycelial morphology of supernatant of HY-88 and HY-91 fermentation broth against *Magnaporthe grisea*.

The supernatant of HY-88 and HY-91 fermentation broth exhibited inhibitory effects on the *Magnaporthe grisea*, with 94.44% and 91.67% inhibition, respectively (Figure 8). The mycelia of the control group were uniform and normal in shape, and the bud tubes were branched and elongated without malformation at the germination point. After the treatment with the supernatant of HY-88 and HY-91 fermentation broth, the hyphae were enlarged and uneven in thickness. In addition, some hyphae were swollen at the top to form a spherical shape, and their growth was seriously hindered.

### 3.8. Tolerance Test of the HY-88 and HY-91

**Figure 9:**
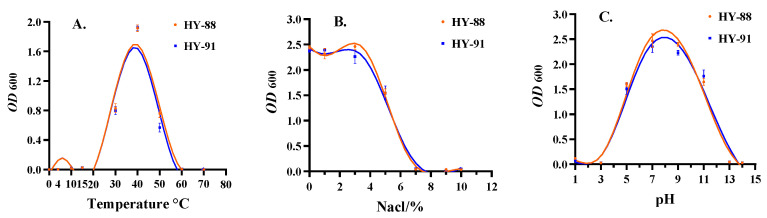
Tolerance test of HY-81 and HY-91. (**A**): Temperature test; (**B**): NaCl tolerance; (**C**): pH test. Assays were performed in triplicate, and the data were reported as mean ± standard deviations.

The temperatures tested ranged from 4 to 50 °C, with seven intermediate points. The HY-88 and HY-91 strains can grow at 10–40 °C, but not at 4 °C and 50 °C (Figure 9A).

Both HY-88 and HY-91 could grow in the concentration range of 1–10% NaCl. However, with the increase in NaCl concentration, both strains grew slowly. HY-88 was more salt tolerant than HY-91, but no significant difference was detected. When the concentration of NaCl was 7–10%, HY-88 and HY-91 almost failed to grow (Figure 9B).

Both HY-81 and HY-91 could not live in highly acidic or alkaline environments. The survival range was between pH 3-13, and the best range was between pH 5 and 11 (Figure 9C).

## 4. Discussion

In recent years, research regarding marine microorganisms has developed rapidly. As an enormous treasure trove of natural resources, the development and utilization of more culturable marine microbial resources is one of the directions of marine microbial research [37].

In this study, a total of 200 culturable strains of 72 different bacteria were obtained by gene sequencing analysis. Bacilli (59.72%) and Gammaproteobacteria (20.83%) were the dominant classes. When studying the diversity of culturable bacteria in the sedimentary environment of the South China Sea, Liu [38] also found that the predominant groups were phylum Firmicutes (54.5%) and Proteobacteria (28.3%). However, Li [39,40] found that α-Proteobacteria and actinomycetes were dominant groups in the surface seawater of the northern South China Sea. We found that *Bacillus* was the dominant bacterial group in the water samples of three different depths in the shallow waters of the South China Sea. And at 2–4 m depth, the diversity of flora is most abundant. This indicated that there were differences in the diversity and quantity of culturable bacteria isolated at different locations and depths of the South China Sea.

The discovery of secondary metabolites based on lipopeptide antibiotics, antimicrobial proteins, polyketo synthetase (PKS) and non-ribosome synthetase (NRPS) gene cluster analysis is of great significance for the discovery of new natural active substances, e.g., a new antifungal peptide with potential application in the biocontrol of plant diseases. [41,42,43]. In this study, we identified that HY-88 could amplify all the fat peptide antibiotics and PKS genes, while HY-91 did not amplify the lipid synthesis peptide antibiotics ituD gene fragments. The difference in these functional genes led to the difference in antifungal function, which requires further study.

The marine bacteria HY-88 and HY-91 screened in this study had a good antifungal effect on the tested plant pathogenic fungi. The fermentation liquid can inhibit several different plant diseases, especially the supernatant of the fermentation broth on rice blast bacteria. The fungistatic rate of the fermentation broth supernatant of the two strains was quite high, exceeding 90%. This indicated that the fungistatic substances of these two strains are secretory. Similarly, some hyphae appeared to swell at the top, or they were broken, their cell wall was thinned, they were partially ruptured and their growth was seriously hindered. A preliminary exploration was performed for the study of the antifungal mechanism of marine culturable bacteria and their antifungal active substances. Moreover, the biocontrol efficacy of strains against *Colletotrichum viniferum* was assayed on detached grape fruit. Our next focus will be the separation and purification of active substances and the analysis of active ingredients. Wen et al. optimized the fermentation conditions of the strain, and the results indicated that the antagonism of the strain on pathogenic bacteria depended on the bacteria itself and the appropriate growth environment [44].

In recent years, the potential biological strains are considered to inhibit phytopathogens. Epinecidin-1, a marine antifungal peptide, interacted directly with *Botrytis cinerea* genomic DNA and delayed gray mold in postharvest peaches. Its exposures induced accumulation of intracellular ROS and increased the permeability of cell membranes, resulting in leakage of nucleic acids and aberrant cell morphology [45]. The volatile organic compounds (VOCs) produced by *Trichoderma atroviride* IC-11 can almost completely inhibit *B. cinerea* growth in vitro. The most abundant volatile compound was 6-pentyl-α-pyrone (6PP), and the binding of VOCs to the surface of hyphae caused their vacuolation to antifungal activity [46]. Kakar [47] found that Bk1, P1 and Bk7 were able to significantly improve plant growth and increase the expression of major defense-related rice genes. In addition, these strains were able to form biofilms and carry multiple lipopeptide biosynthetic genes as revealed, and unanimously suppressed the mycelial growth of antifungal activities against *Magnaporthe oryzae*, rice sheath blight diseases. Some *Bacillus subtilis* biocontrol strains have better control effects on rice bacterial diseases. For example, the strain RH5 significantly increased plant growth and triggered resistance in rice plants through the production of defense-related antioxidant enzymes [48]. Four *Bacillus* isolates BKOU-1, BKOU-8, BKOU-13 and BKOU-14, showed significant plant growth-promoting traits, hydrolytic enzyme production and effectively restricted the mycelial growth and developed resistance to *Fusarium oxysporum* and *Macrophomina phaseolina* [49]. *Bacillus agave* JN-369 showed antagonistic effects on a variety of phytopathogens. *B. amyloliquefaciens* B4 shows prominent antifungal activity against *P. expansum* [50]. The cyclic lipopeptide (CLP) iturin A was isolated from the fermentation broth of *Bacillus velezensis* 11-5, which showed antagonistic properties against the isolates of blast pathogen oryzae [24]. *Bacillus* strains have been proven to function in field assays [51]. Bacillus WJ-1 could significantly inhibit the hyphal growth of plant pathogenic fungi such as *Fusarium graminearum*, *Chrysostachys nicotiana*, *Sclerotinia brassica* and *Sclerotinia brassica* [52]. The inhibition mechanism of these strains against phytopathogens laid the foundation for our later research on antagonistic mechanisms.

In the tolerance test of the strains in this study, there was an important influence on fungal growth [53]. The medium and fermentation conditions we used did not reach the optimal growth conditions of the strains; thus, the antagonistic effects of these two strains on the tested pathogenic strains may not be optimal. Therefore, studying the tolerance of strains lays the foundation for our next step in determining the optimal culture conditions for active substances. Incubation temperature and time generally have an important influence on fungal growth.

## 5. Conclusions

This study revealed the diversity of culturable bacteria in marine water samples from the South China Sea and screened two strains with obvious fungistatic functions. The above research results provide a research basis for the next step to develop new fungistatic agents with biological control functions.

## Figures and Tables

**Table 2:** Inhibition rates of the HY-88 and HY-91 strains to plant pathogens.

Fungistatic Rate (%)	*Botrytis cinerea*	*Fusarium oxysporum*	*Colletotrichum viniferum*	*Magnaporthe grisea*
HY-88	51.72%	54.29%	52.17%	90.91%
HY-91	34.48%	48.57%	47.83%	86.36%

## Data Availability

Data are available by request through the authors.
